# Why do men seek mental health treatment less than women? An analysis of a Hungarian community sample

**DOI:** 10.1192/j.eurpsy.2026.10762

**Published:** 2026-07-31

**Authors:** V. Swisher, D. Őri, R. Wernigg

**Affiliations:** 1 Psychology, Penn State University, State College, United States; 2 Institute of Behavioral Sciences, Semmelweis University; 3 National Directorate-General for Hospitals, Budapest, Hungary

## Abstract

**Introduction:**

Research suggests that men are less likely than women to seek psychological treatment. Identifying barriers to mental health treatment seeking among men may help identify intervention targets.

**Objectives:**

The present study aimed to examine demographic, clinical, and stigma-related variables associated with treatment seeking, identify those that differ between genders, and explore whether these variables statistically account for the relationship between gender and treatment seeking.

**Methods:**

Participants (*N* = 345; 62% female; M_age_ = 46.37) were recruited from primary care offices across Hungary and completed a survey assessing demographics, lifetime treatment seeking, self and public stigma, anxiety, depression, and experiential avoidance. Independent samples *t*-tests were used to examine gender differences in study variables. Binary logistic regression was conducted to identify predictors of lifetime treatment seeking, and indirect effects were tested using mediation models with bootstrapped confidence intervals.

**Results:**

As expected, gender significantly predicted lifetime treatment seeking, *B* = 0.82, SE = 0.34, Wald = 5.88, *p* = .015, OR = 2.26, 95% CI [1.17, 4.37], with women more likely to have sought treatment. Additionally, attitudes toward seeking help were positively associated with lifetime treatment seeking, *B* = 0.12, SE = 0.03, Wald = 10.07, *p* = .002, OR = 1.13 95% CI [1.05, 1.22], such that more positive attitudes were linked to a higher likelihood of having sought treatment. Independent samples *t*-test revealed that men and women did not significantly differ in age, public stigma, depression, or experiential avoidance, *p*s = .156–.535. However, men had significantly greater self-stigma, less positive attitudes toward seeking help, higher income, and lower anxiety relative to women, *ps* = .003-.019. Analyses of indirect effects revealed that both attitudes toward help-seeking and anxiety partially mediated the association between gender and treatment seeking. Specifically, men’s less positive attitudes toward help-seeking were associated with lower odds of treatment seeking (indirect effect = 0.14, boot SE = 0.06, 95% CI [0.03, 0.29]), and women’s higher anxiety scores were associated with greater odds of treatment seeking (indirect effect = 0.20, boot SE = 0.09, 95% CI [0.04, 0.39]). In both cases, the direct effect of gender on treatment seeking remained significant, indicating partial mediation.

**Conclusions:**

Findings indicate that men were less likely than women to report lifetime treatment seeking. Mediation analyses suggested this association was accounted for, in part, by differences in anxiety and attitudes toward seeking help. Improving treatment seeking attitudes in men may be a relevant intervention target. This may be especially important given that men did not differ from women in other relevant clinical variables, including depression and experiential avoidance.

**Disclosure of Interest:**

None Declared

